# Nonalcoholic Fatty Liver Disease: Focus on Lipoprotein and Lipid Deregulation

**DOI:** 10.1155/2011/783976

**Published:** 2011-07-02

**Authors:** Klementina Fon Tacer, Damjana Rozman

**Affiliations:** Center for Functional Genomic and Biochips, Institute of Biochemistry, Faculty of Medicine, University of Ljubljana, Zaloška 4, 1000 Ljubljana, Slovenia

## Abstract

Obesity with associated comorbidities is currently a worldwide epidemic and among the most challenging health conditions in the 21st century. A major metabolic consequence of obesity is insulin resistance which underlies the pathogenesis of the metabolic syndrome. Nonalcoholic fatty liver disease (NAFLD) is the hepatic manifestation of obesity and metabolic syndrome. It comprises a disease spectrum ranging from simple steatosis (fatty liver), through nonalcoholic steatohepatitis (NASH) to fibrosis, and ultimately liver cirrhosis. Abnormality in lipid and lipoprotein metabolism accompanied by chronic inflammation is the central pathway for the development of metabolic syndrome-related diseases, such as atherosclerosis, cardiovascular disease (CVD), and NAFLD. This paper focuses on pathogenic aspect of lipid and lipoprotein metabolism in NAFLD and the relevant mouse models of this complex multifactorial disease.

## 1. Introduction

Nonalcoholic fatty liver disease (NAFLD) is progressively diagnosed worldwide and is considered to be the most common liver disorder in Western countries, estimated to affect at least one-quarter of the general population [1, 2]. NAFLD used to be almost exclusively a disease of adults but is now becoming a significant health issue also in obese children. The prevalence of childhood obesity has significantly increased over the past three decades [3, 4] and boosted the prevalence of NAFLD in adolescents (reviewed in [5]). 

NAFLD covers a spectrum of hepatic pathologies, ranging from simple steatosis to nonalcoholic steatohepatitis (NASH). It strongly associates with obesity, insulin resistance, hypertension, and dyslipidaemia and is now regarded as the liver manifestation of metabolic syndrome [6]. Simple steatosis is largely benign and nonprogressive whereas NASH is characterized by hepatocyte injury, inflammation, and fibrosis and can lead to cirrhosis, liver failure, and hepatocellular carcinoma [7]. 

Lipid accumulation in the liver is the major hallmark of NAFLD. A comprehensive understanding of the mechanisms leading to liver steatosis and further transition to nonalcoholic steatohepatitis (NASH) still remains elusive. There is no simple solution to understand the multi-factorial nature of NAFLD appearance and progression, presumably due to the nonlinear interactions of those factors. Abnormalities in lipid and lipoprotein metabolism accompanied by chronic inflammation are considered to be the central pathway for the development of several obesity-related co-morbidities such as NAFLD and cardio-vascular disease (CVD) [8, 9]. 

NAFLD is not an innocent bystander in the metabolic syndrome. Rather, it represents an important self-governing risk for the development of CVD. In NAFLD patients, liver overproduces several atherogenic factors, such as cytokines and “bad” lipoproteins. In this manner, fatty liver is associated with increased serum low-density lipoproteins (LDL) and triglycerides, combined with decreased high-density lipoproteins (HDL) that represent a threat for CVD development (reviewed in [10]). There are many evidences suggesting that NAFLD is linked to the increased incidence of CVD, both in nondiabetic and type 2 diabetic patients [11]. Furthermore, a prospective observational study of Hamaguchi et al. implied that NAFLD may play a central role in the cardiovascular risk of metabolic syndrome [12]. NAFLD is also very common in type 1 diabetes and is strongly associated with increased prevalence of CVD independent of other confounding factors [13]. In addition to the liver-related causes, CVD represents the major survival risk of patients with NASH [14]. However, the nature of the relationship NAFLD/CVD is still under debate. McKimmie and coauthors [15] did not find independent association between hepatic steatosis and CVD in a subset of participants in Diabetes Heart Study. They suggested that hepatic steatosis is more a secondary phenomenon than a direct mediator of CVD. Even so, sufficient evidence exists that CVD risk assessment seems mandatory in NAFLD patients. 

**Figure 1:**
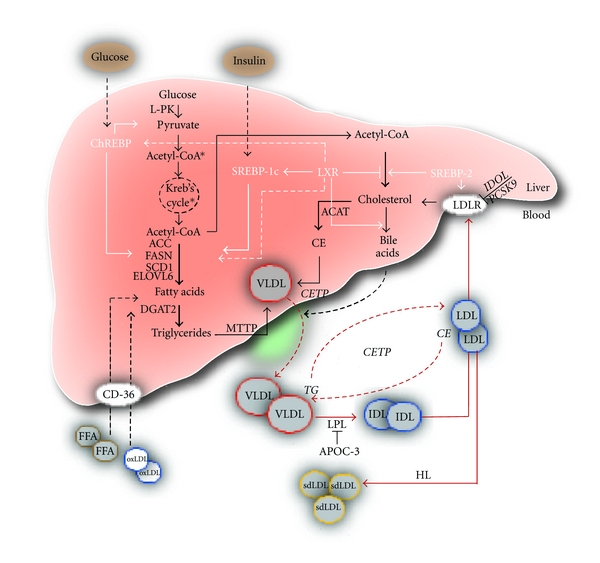
Lipid and lipoprotein pathways in the pathogenesis of NAFLD. NAFLD is considered to be liver manifestation of obesity and metabolic syndrome. In response to insulin and glucose, transcription factors SREBPs and ChREBP are activated and induce the expression of genes involved in the synthesis of fatty acids and cholesterol in the liver. Enhanced lipogenesis leads to enhanced VLDL production, a major metabolic perturbation in NAFLD. Increased VLDL secretion in plasma results in increase in LDL through CETP-mediated exchange of cholesteryl esters and triglycerides between LDL and VLDL, followed by triglyceride removal from LDL by hepatic lipase (HL). Liver removes LDL from circulation by LDLR-mediated endocytosis. Oxidized LDL and FFA are transported to the liver by CD-36, FA translocase, and scavenger receptor. Italics: metabolic genes, black lines: metabolic pathways, black dash lines: coming out or in the liver, white lines: transcriptional regulation, *process in the mitochondria.

**Figure 2:**
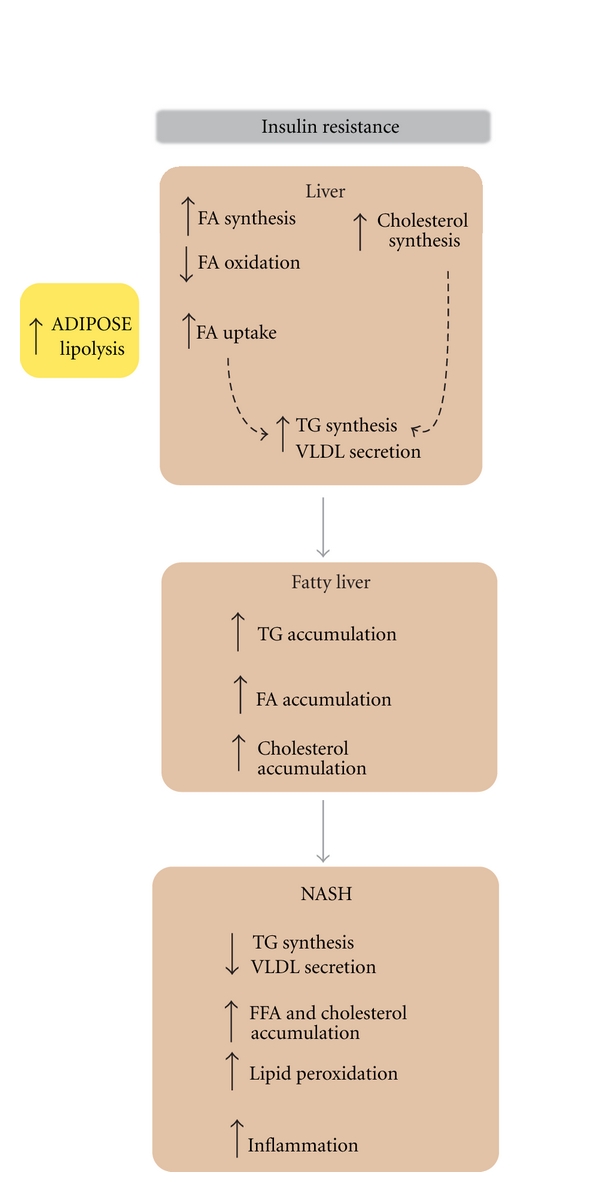
Summary of insulin resistance-induced lipid abnormalities and consequent pathogenesis of NAFLD. In an insulin resistant setting, insulin is unable to inhibit lipolysis in adipose tissue leading to overflow of free FA into the bloodstream and in the liver. In the liver, hyperinsulinemia and hyperglycemia induce the synthesis of fatty acid and cholesterol which results in increased triglyceride synthesis and VLDL assembly and secretion. Since triglyceride synthesis prevails over VLDL secretion, excess triglycerides accumulate and lead to fatty liver development. In NASH, triglyceride synthesis and VLDL assembly is impaired and free FA and cholesterol accumulate. Increased lipid accumulation leads to lipid peroxidation and inflammation which exacerbates liver damage.

NAFLD pathogenesis as a two-hit model was initially proposed by Day and James [16]. First, insulin resistance causes lipid accumulation in hepatocytes; second, cellular insults such as oxidative stress, lipid oxidation, and inflammation result in NASH. Deregulation of fat metabolism in the fatty liver is accompanied by overproduction of very-low-density lipoproteins (VLDL), the characteristic lipoproteins of the metabolic syndrome [17]. LDL has recently attracted attention since small, dense LDL is the most atherogenic subclass of LDL, and this subclass is elevated in metabolic syndrome and fatty liver. However, elevated VLDL is likely the key metabolic disturbance and correlates strongly with obesity and metabolic syndrome. Fatty liver-associated dyslipidemic profile characterized by large VLDL, small dense LDL, and decreased large HDL correlates with the intrahepatic lipid content. Herein, we review recent understanding of lipid and lipoprotein homeostasis in the development of NAFLD and the relevant polygenic mouse mode that help in unraveling the pathogenesis of this disease. The studies included in this review paper (31 clinical and 82 experimental studies) were selected based on the involvement in lipid and lipoprotein metabolism and reported association with NAFLD and insulin resistance. Major pathways of lipid and lipoprotein homeostasis relevant for the development of NAFLD are depicted in Figure 1.  Figure 2 summarizes the association between insulin resistance-induced lipid abnormalities and pathogenesis of NAFLD.  Table 1 recapitulates the physiologic role of all receptors and enzymes described in the paper and the association with insulin resistance and the pathogenesis of NAFLD. Finally, we briefly discuss how reviewed pathways are involved in the therapeutic strategies for NAFLD.

## 2. Lipoprotein Metabolism in NAFLD 

Lipid transport in plasma utilizes highly specialized lipoprotein complexes. After a meal, dietary fat and cholesterol are absorbed into intestinal cells and incorporated in nascent chylomicrons. The liver is another important source of lipoproteins. A central metabolic role of the liver is to maintain plasma glucose within narrow physiological limits regardless of the nutritional state of the animal. In energy excess, glucose is converted to fatty acids, which are further used to synthesize triglycerides. Triglycerides can be stored as lipid droplets within hepatocytes or incorporated into very-low-density lipoproteins (VLDL) and secreted into the blood. Once in the blood, triglyceride content of these particles is progressively reduced by the action of lipoprotein lipase (LPL), eventually resulting in intermediate-density lipoproteins (IDLs) and low-density lipoproteins (LDL) with relatively high cholesterol content [69]. LDL circulates and is absorbed by the liver by binding of LDL to LDL receptor [70]. 

Patients with insulin resistance increase VLDL secretion as they attempt to maintain hepatic lipid homeostasis. Therefore, insulin resistance is associated with abnormal concentration of lipoproteins [71, 72], elevated VLDL production, and increase in plasma LDL [73]. Elevated plasma LDL was also found in patients with NAFLD [5, 74]. 

Association of fatty liver and small dense LDL (sdLDL) concentration is now well documented [75, 76]. As the triglyceride-rich VLDL is entering plasma at an accelerated rate, small, dense LDL, the most atherogenic subclass of LDL, develop after triglycerides are gradually removed from LDL. Two enzymes are implicated in this process. First, cholesteryl ester transfer protein (CETP) [18] facilitates the transfer of triglycerides from VLDL to LDL (and cholesteryl esters from LDL to VLDL), and, second, hepatic lipase increases lipolysis of triglyceride-rich LDL resulting in the formation of sdLDL [21]. Thus, CETP remodels VLDL in circulation, enriches it in cholesterol, and also favors, together with HL, the formation of sdLDL. CETP activity is increased in hepatic steatosis patients [19]. LDL receptor shows a lower affinity for smaller particles, therefore such particles stay longer in the circulation [77]. Hyperlipidemia can be further exacerbated by low activity of lipoprotein lipase, or by high level of apolipoprotein C-3 (APOC-3), an inhibitor of lipoprotein lipase. Indeed, APOC-3 polymorphisms have been associated with fatty liver in humans [20]. However, this association was not found in recent Dallas Heart Study [22]. 

In contrast to simple steatosis, the steatohepatitis links to dysfunctional VLDL synthesis and secretion [78]. Fujita et al. [78] investigated the difference in lipid metabolite and serum lipoprotein levels between patients with steatosis or NASH. Hepatic lipid profiles in the two patient groups were similar; however, VLDL synthesis and export were impaired in NASH. This is in line with the two main experimental animal models that are often used to study NAFLD [79]. A high-fat/high-calorie diet is a generalized fatty liver model [79]. A choline-deficient/1-amino acid-defined (CDAA) diet, which disturbs VLDL secretion is an NASH model [80]. The former involves only fatty liver whereas the latter includes also steatohepatitis and liver cirrhosis [81]. 

The blockade of hepatic VLDL secretion results in accumulation of triglycerides in the liver. Microsomal triglyceride transfer protein (MTTP) is essential for the formation of VLDL in the liver [24]. Mice that cannot secrete VLDL due to the conditional knockout of *Mttp* in the liver exhibit markedly reduced levels of triglycerides in the plasma and develop hepatic steatosis [82, 83], however, without insulin resistance and inflammation [84]. In line with the rodent data, human MTTP polymorphisms lead to decreased MTTP activity and VLDL export and are associated with greater intracellular triglyceride accumulation. Altogether, this impacts NASH pathogenesis [25], possibly through modulating postprandial lipid profile [26]. On the other hand, a high-fat diet was shown to induce the methylation of MTTP and consequently reduce its mRNA level [85]. The postprandial phase has been linked to increased oxidative stress [86], and increased lipid peroxidation is implicated in NASH pathogenesis. Oxidized LDL can activate hepatic stellate cells that are crucial in NASH pathogenesis [87].

Sortilins, intracellular sorting receptors for apolipoprotein B 100 (APO-B 100), are new players in lipoprotein metabolism. Genome-wide association studies (GWAS) of common genetic variations associated sortilin 1 (*Sort 1*) gene with LDL metabolism. Several findings provide evidences that sortilin 1 is involved in the hepatic metabolism of lipoproteins containing APO-B, although the precise mechanism waits for further elucidation. Kjolby et al. showed that Sort 1 interacts with APO-B 100 in the Golgi and facilitates the formation and hepatic export of VLDL lipoproteins, thereby regulating plasma LDL concentration. They showed that *Sort 1* overexpression stimulated hepatic release of lipoproteins and increased plasma LDL [27]. Using different mouse models, Musunuru et al. [28] showed an inverse relationship between *Sort1 1 *expression and circulating LDL cholesterol. Namely, liver-specific overexpression of *Sort 1* in mice decreased serum LDL cholesterol whereas knockdown had opposite effect. Moreover, Linsel-Nitschke showed that Sort 1 enhanced LDL endocytosis [29]. Further studies are needed to understand the mechanistic link between sortilin 1 and lipoprotein metabolism. 

Plasma levels of LDL, the major cholesterol carrying lipoprotein in humans, are determined by the relative rates of production and clearance by LDL receptor. The groundbreaking work of Goldstein and Brown defined the pathway of LDL receptor-mediated endocytosis and its regulation by cholesterol-dependent negative feedback transcriptional regulation (reviewed in [88]). In addition to transcriptional regulation, LDLR is also regulated on the protein level. PCSK9 (proprotein convertase subtilisin/kexin type 9) enhances LDLR degradation, resulting in low-density lipoprotein accumulation in plasma. PCSK9 binds the EGF-A domain of the low-density lipoprotein receptor (LDLR) and favors the targeting of the LDLR to endosomes/lysosomes and its degradation (reviewed in [30]). Individuals with loss-of-function mutations in PCSK9 have reduced plasma levels of LDL cholesterol and are protected from CHD [89]. Inhibiting the action of PCSK9 on the LDLR has emerged as a novel therapeutic target for hypercholesterolemia. It was shown recently that PCSK9 deficiency confers resistance to liver steatosis; however, this effect seemed to be LDLR independent [31].

## 3. Cholesterol and Triglyceride Homeostasis in NAFLD

Abnormal lipoprotein concentration in plasma reflects disturbances in homeostasis of major lipid components of lipoproteins, triglycerides, cholesterol, and cholesterol esters. Excessive accumulation of triglycerides in the liver is the hallmark of NAFLD. The potential sources of fat contributing to hepatic steatosis are dietary fatty acids through uptake of intestine-borne chylomicron remnants, increased lipolysis of peripheral fat store, and *de novo* synthesis. Tracer studies in obese humans with NASH demonstrated that 60% of triglycerides in the liver arose from free fatty acids, 25% from *de novo* lipogenesis, and 15% from the diet [90].

### 3.1. Fatty Acids and Triglycerides

Insulin resistance is associated with deregulation of adipose-derived fatty acid flux in the fasting state [91]. In NAFLD patients, insulin does not suppress adipose tissue lipolysis to the same extent that it does in healthy individuals [32]. Studies on mice have revealed that hormone-sensitive lipase- (HSL-) knockout mice show increased insulin sensitivity with decreased hepatic triglyceride content [33]. Fatty acid uptake was believed to be predominantly passive; however, this concept has been challenged by the discovery of cluster differentiation protein-36 (CD-36), fatty acid translocase [34], and its association with NAFLD. CD-36 is regulated by insulin and can induce hepatosis [92]. In NASH, development of disease is associated with increased expression of CD-36 [35–37]. 

Among major causes of fat accumulation in NAFLD is the inability of the liver to regulate the changes in lipogenesis in the transition from fasted to fed state. Several studies suggested increased hepatic lipogenesis in hepatic steatosis. Increased lipogenesis may have dual effect: increased triglyceride synthesis and decreased fatty acid oxidation through production of malonyl-CoA [93], both leading to increased triglycerides content in fatty liver. Sanyal et al. reported that *β*-oxidation of fatty acids in the liver was increased in patients with NASH [94]. However, this increase might not sufficiently overcome the elevated rates of hepatic fatty acid synthesis.

*De novo* fatty acid synthesis is the metabolic pathway converting excess carbohydrates into fatty acids, which are ultimately esterified to form triglycerides. Several indications show an increase in fatty acid synthesis in NAFLD. Humans [95] and mice [96] with fatty liver accumulate oleic acid, the end product of fatty acid synthesis, indicating increase in this pathway. Fatty acid synthase (FASN) catalyzes the last step in fatty acid biosynthesis, and thus, it is believed to be a major determinant of the maximal hepatic capacity to generate fatty acids by *de novo* lipogenesis. The expression of FASN mRNA in human liver is higher in hepatic steatosis [41]. Further on, knocking down several genes of the fatty acid synthesis, desaturation, and elongation, such as *Acc*, *Scd1*, and *Elovl6*, reversed several metabolic defects associated with hepatic steatosis in experimental animals [42–46]. NAFLD is also associated with depletion of n-3 polyunsaturated fatty acids (PUFA), a consequence of decreased liver Δ-6 and Δ -5 desaturase in obese NAFLD patients ([47], reviewed in [48]).

The mouse strain C57BL/6 is one of the most frequent laboratory strains for studies of high lipid-related metabolic disorders. The strain has a long life period and a high pre-disposition for development of induced hyperlipidemias. Hepatic lipid metabolic processes are circadian in this strain [97]. An 8-week hyperlipidemic diet (1.25% cholesterol, 0.5% cholic acid, 15% fat) leads to fatty liver [98, 99], and a 5-week atherogenic diet to NASH [100]. This mouse strain has been successfully applied also in studies of cholesterol-lowering effects of atorvastatin [101]. 

Bübger et al. developed a unique polygenic model of NAFLD—so-called “fat” mice [102]. Genetic studies have confirmed a typical polygenic basis of this obesity [103–105]. In contrast to many other animal models of obesity and NAFLD, this line develops NAFLD already on a low-to-moderate food intake, and development of later stages of NAFLD is accelerated on high-fat diet [106, 107]. Plasma and liver lipid profiles are perturbed similarly as in NAFLD patients. Liver transcriptome exhibits dramatic changes with perturbations in genes of *de novo* cholesterol synthesis, bile and glucose metabolism, liver receptors, and immune response [107]. This polygenic model is unique since it allows studies of the genetic and molecular mechanisms of both early hepatic fat accumulation and advanced forms of NAFLD. Other mouse models with defects in cholesterol synthesis might also represent useful tools for understanding NAFLD [108]. 

Insulin and glucose both drive lipogenesis [109] by respective transcription factors, SREBP-1c (sterol regulatory element binding protein 1c), and ChREBP (carbohydrate regulatory element binding protein). Hyperinsulinemia despite insulin resistance induces the expression of SREBP-1c, a transcriptional activator of all lipogenic enzymes, resulting in increased rate of fatty acid synthesis [49, 50]. Overexpression of *Srebp-1c* in transgenic mouse livers leads to classic fatty liver due to increased lipogenesis [110]. In contrast, inactivation of *Srebp-1* gene in ob/ob mice results in 50% reduction of triglycerides in these mice [111]. SREBP-1c also activates acetyl-coA carboxylase 2 (Acc2) [53] that produces malonyl-CoA. Increase in malonyl-CoA results in decreased fatty acid oxidation due to inhibition of carnitine palmitoyl transferase-1 (Cpt-1), which shuttles fatty acids into mitochondria [54]. Indeed, *Acc2* knockout mice are resistant to obesity with increased Cpt-1 activity and consequent fatty acid oxidation [112].

In addition to insulin, glucose activates lipogenesis through transcriptional factor ChREBP. ChREBP simultaneously activates liver-type pyruvate kinase (L-PK), key regulator of glycolysis and all lipogenic genes [113, 114], including acetyl-CoA carboxylase and fatty acid synthase [115]. *ChREBP* gene expression and nuclear protein content are significantly increased in liver of ob/ob mice. Liver-specific inhibition of *ChREBP* improves hepatic steatosis and insulin resistance in ob/ob mice [51]. Bricambert et al. [52] went on and showed upstream regulation of ChREBP. Serine/threonine salt-inducible kinase 2 (SIK2) directly regulates hepatic lipogenesis through the inhibition of p300 acetylation of *ChREBP*, which in turn increases ChREBP-induced transcription. Inhibition of hepatic p300 thus offers a novel target for treating hepatic steatosis.

Triglyceride accumulation in hepatocytes was considered to be the major pathogenic trigger in the development of NAFLD. Diacylglycerol acyltransferase 2 (DGAT2) catalyzes the final step in hepatocyte triglyceride biosynthesis. Suppression of DGAT2 reversed diet-induced hepatic steatosis and insulin resistance [55] as well as attenuates hyperlipidemia [56]. However, recent findings suggest that triglyceride synthesis *per se* may not be harmful to hepatocytes. Rather, it provides a useful mechanism for buffering free fatty acid accumulation [57]. Yamaguchi et al. [57] show that inhibiting triglyceride synthesis by inhibiting DAGT2 does improve hepatic steatosis, yet it exacerbates liver damage and fibrosis in obese mice with nonalcoholic steatohepatitis. Lipotoxicity arises when hepatic triglyceride synthesis is unable to accommodate increased free fatty acid accumulation. Thus, rather than being hepato-toxic, liver triglyceride accumulation is actually hepatoprotective in obese, insulin-resistant individuals. 

Hepatic fatty acids are derived from several sources, including adipose tissue lipolysis, chylomicron-TAG lipolysis, and de novo lipogenesis, and can be stored as TAG in lipid droplets located within the cytosol [116]. Hepatic TAG stores are mobilized by several hepatic lipases. Adipose triacylglycerol lipase (*Atgl*) that selectively performs the first step in TAG in the liver is reduced in several rodent models of obesity, and *Atgl* ablation leads to steatosis, although increased TAG content in the hepatocytes from Atgl-deficient mice does not enhance insulin sensitivity [58, 59]. Similarly, inhibiting expression of ATGL coactivator (gene identification-58 (*Cgi-58*)) resulted in a large increase in hepatic TAG content, yet in a decrease in insulin resistance [60]. Further, overexpressing ATGL specifically in the liver of obese mice did decrease liver steatosis, but it only mildly enhanced liver insulin sensitivity [58], suggesting that ATGL might be a pharmacological therapeutic target for NAFLD but not type 2 diabetes.

### 3.2. Cholesterol and Cholesteryl Esters

Cholesterol is either synthesized *de novo *in the liver or delivered to the liver by lipoproteins (reviewed in [117]). The metabolism of cholesterol in NAFLD remains poorly explored. Insulin resistance is associated with increased cholesterol synthesis [118, 119]. Recent metabolomic analysis implicated that cholesterol synthesis in NAFLD patients is increased, in contrast to diminished absorption of cholesterol [120]. The expression of cholestrogenic genes was also found elevated in NAFLD patients, accompanied by decreased SREBP-2 and LDLR expression [23]. In rodents, excess cholesterol intake contributes to the development of NAFLD even in the absence of obesity [121, 122]. 

Cholesterol rich-atherogenic diet induces oxidative stress and provokes inflammation. The transition towards hepatic inflammation is the key factor in NASH pathogenesis and promotes progression to liver damage. Currently, NASH is thought to develop* via *the “two hit” model [16]. According to this hypothesis, hepatic steatosis represents “first hit” and is still reversible. The “second hit” includes NASH progression beyond hepatic steatosis that promotes oxidative stress, inflammation, cell death, and fibrosis [93]. Marí et al. [123] provided evidence that mitochondrial loading of free cholesterol, but not free fatty acids or triglycerides, sensitizes the liver to TNF-*α*-induced steatohepatitis. In line with these results, Wouters et al. [124] found that dietary cholesterol rather than lipid accumulation is an important risk factor for the progression to hepatic inflammation. High-cholesterol diet led to increased VLDL and was sufficient to cause inflammatory response in the liver. As mentioned earlier, increased VLDL and accompanied hypertriglyceridemia underlie the synthesis of small, dense LDL with lower affinity for LDL receptor. Therefore, these particles stay in circulation for longer period and are prone to oxidation [77]. Oxidized LDL can bind scavenger receptors, such as CD-36 [38–40], which are present on Kupffer cells and prompt inflammatory response. 

Inflammatory cytokine TNF-*α* is overexpressed in the liver of obese mice and mediates insulin resistance [125, 126]. Furthermore, TNF-*α* is required for the development of fatty liver and subsequent liver damage by alcohol [127, 128]. We [129, 130] and others have showed that TNF-*α* activates cholesterol synthesis and inhibits cholesterol elimination through bile acids, which together contribute to increase in LDL-cholesterol and reduction of HDL-cholesterol. 

Cholesterol is indispensible, however, toxic in excess. Intracellular level of cholesterol is tightly regulated by a number of mechanisms that govern uptake, synthesis, catabolism, and export. Two master regulators of these pathways are the transcription factors SREBP-2 and LXR (liver X receptor). When intracellular cholesterol levels drop, SREBP-2 induces cholesterol biosynthesis and uptake [61]. In contrast, excess intracellular cholesterol inhibits SREBP-2 and activates LXR, which in turn promotes cholesterol export and elimination [62]. 

LXRs are nuclear receptors that control lipid metabolism. Nuclear receptors may have a crucial role in lipid-related genesis of NAFLD [131, 132]. LXRs were discovered as sterol sensors that regulate cholesterol homeostasis [63, 64]. In rodents, LXR promote cellular cholesterol efflux, transport, and excretion [133]. LXRs have emerged as promising drug targets for antiatherosclerotic therapies. However, pharmacological LXR activation also induces hepatic steatosis and promotes the secretion of VLDL particles by the liver, complicating the clinical application of LXR agonists. Namely, LXR activates SREBP-1c, a master transcriptional regulator of fatty acid synthesis [134, 135]. Moreover, LXR has a central role in insulin-mediated activation of SREBP-1c-induced fatty acid synthesis in liver [136]. In addition, LXR can also promote lipogenesis in an SREBP-1c-independent manner [137, 138] and activate other lipogenic transcriptional factor ChREBP [139]. Further on, LXR was also shown to induce the expression of CD-36, fatty acid transporter, and scavenger receptor, suggesting another mechanism by which LXR can promote fatty liver [92]. LXR also exerts negative control of LDLR-mediated cholesterol uptake by inducing the expression of Idol (inducible degrader of the LDLR). Idol catalyzes the ubiquitination of LDLR and targets it for degradation [65].

Intracellular free cholesterol is converted into cholesteryl ester by acyl-Coenzyme A: cholesterol acyltransferase (ACAT). The function of ACAT2 in the hepatocyte is to provide esterified cholesterol for incorporation into very-low-density lipoprotein (VLDL), as well as to provide cholesteryl ester for cytoplasmic lipid droplets, a means for storage when liver cholesterol is abundant. Increased VLDL cholesteryl ester secretion occurs in livers of monkeys fed dietary cholesterol (reviewed in [140]). Mice, genetically engineered to lack *Acat2* in both the intestine and the liver, were dramatically protected against hepatic neutral lipid (TG and cholesteryl ester) accumulation, in particular with elevated cholesterol diet. Inhibition of hepatic Acat2 can prevent dietary cholesterol-driven hepatic steatosis in mice [66].

Recently, Niemann-Pick C1-like 1 (NPC1L1) has been shown to play a pivotal role in cholesterol absorption [67]. Unlike mouse NPC1L1 protein that is predominantly expressed in the intestines, human and rat NPC1L1 is also abundantly expressed in the liver. Loss of NPC1L1 expression has been shown to protect against diet-induced fatty liver [68].

## 4. Conclusion

Nonalcoholic fatty liver disease (NAFLD) is a multi-factorial disorder with contribution of a variety of genetic and environmental factors that up till now no effective treatments exist for them. By poorly defined mechanisms, including free fatty acid and cholesterol accumulation accompanied by oxidative stress and inflammation, NAFLD may progress to the irreversible steatohepatitis (NASH) and further to cirrhosis or hepatocellular carcinoma. Herein, we review the major advances in the understanding of the pathogenic aspect of lipid and lipoprotein metabolism in NAFLD that could affect future therapeutic strategies. Currently, the only established treatment is weight loss since obesity underlies insulin resistance and NAFLD (reviewed in [141]). Caloric restriction reverses hepatic insulin resistance and steatosis in rats [142]. Fasting inhibits cholesterol and fatty acid synthesis and has protective effect on lipid metabolism [129]. Further, two different obesity-treatment drugs are currently on the market: orlistat, which reduces intestinal fat absorption, and sibutramine, an appetite suppressant (reviewed in [141]). 

NAFLD and cardiovascular disease share common risk factors, in particular disturbed lipid homeostasis accompanied by dyslipidemia. In patients with NAFLD, inhibition of cholesterol synthesis by statins alone [143] or in combination with antioxidants was shown beneficial [144]. However, the statin therapy shares common risks of drug failure in some individuals that can develop hepatoxicity or drug interactions [145]. Further on, inhibition of SREBP pathway represents another potential treatment for NAFLD. Inhibition of SREBP by a small molecule, betulin, decreased the biosynthesis of cholesterol and fatty acid [146]. Recent reports suggest a potential benefit of inhibiting intestinal cholesterol absorption by ezetimibe NPC1L1-inhibitor [147, 148]. Fenofibrates were also shown to decrease cholesterol absorption at the level of intestinal NPC1L1 expression [149]. 

Due to the complex multi-factorial nature of the disease, combined treatment may be needed to achieve better results. Indeed, dual inhibition of cholesterol absorption and synthesis and coadministration of ezetimibe/simvastatin offer a highly efficacious lipid-lowering strategy [150] and were shown to be effective and safe also in NAFLD patients [151].

In the complex pathology of NAFLD/NASH, the integrative approaches focusing on networks rather than on individual molecules, and applying environmental perturbations (diet, drugs, rhythm, etc.) in suitable animal models, can represent new venues towards predictive markers and successful therapies.

## Figures and Tables

**Table 1:** The physiologic role of all receptors and enzymes described in the paper and their association with insulin resistance and pathogenesis of NAFLD.

Gene symbol	Name	Physiological role	Insulin resistance	Deregulation in NAFLD	Loss-of-function models and NAFLD	Polymorphisms associated with NAFLD	Reference
*CETP*	Cholesteryl ester transfer protein	Facilitates the transfer of TG from VLDL to LDL and CE from LDL to VLDL	Increased	Increased		+	[18–20]
*HL*	Hepatic lipase	Facilitates the clearance of TG from VLDL, increases formation of sdLDL	Increased				[21]
*LPL*	Lipoprotein lipase		Decreased	Decreased			[20]
*APOC-3*	Apolipoprotein C-3	Inhibitor of lipoprotein lipase	Increased	Increased			[20, 22]
*LDLR*	LDL receptor	LDL intake		Decreased			[23]
*MTTP*	Microsomal triglyceride transfer protein	Formation of VLDL in the liver		Decreased	Develop NAFLD	+	[24–26]
*SORT 1*	Sortilin 1	Intracellular sorting receptors for apolipoprotein B100 lipoproteins				+	[27–29]
*PCSK9*	Proprotein convertase subtilisin/kexin type 9	Enhances LDLR degradation			Resistance		[30, 31]
*HSL*	Hormone-sensitive lipase	Adipose tissue lipolysis	Increased	Increased	Resistance		[32, 33]
*CD-36*	Fatty acid translocase; scavenger receptors	FA uptake; oxidized LDL uptake	Increased	Increased	Protective		[34–40]
*FASN*	Fatty acid synthase	Catalyzes the last step in fatty acid biosynthesis		Increased	Protective		[41]
*ACC*	Acetyl coenzyme A (acetyl-CoA) carboxylase	Fatty acid biosynthesis			Protective		[42, 43]
*SCD1*	Stearoyl-CoA desaturase	Fatty acid desaturation			Protective		[44, 45]
*ELOVL6*	Elongation of long-chain fatty acids	Fatty acid elongation			Protective		[46]
*FADS1 FADS2*	Δ-5 and Δ-6 desaturase	PUFA desaturation	Decreased				[47, 48]
*SREBP-1c*	Sterol regulatory element binding protein 1c		Increased	Increased	Protective		[49, 50]
*ChREBP*	Carbohydrate regulatory element binding protein		Increased	Increased	Protective		[51]
*SIK2*	Serine/threonine salt-inducible kinase 2	Increases ChREBP			Protective		[52]
*CPT-1*	Carnitine palmitoyl transferase-1	Shuttles fatty acids into mitochondria, fatty acid oxidation	Decreased				[53, 54]
*DGAT2*	Diacylglycerol acyltransferase 2	Triglyceride biosynthesis	Increased	Increased/decreased in NASH	Improves steatosis, aggravates NASH		[55–57]
*ATGL*	Adipose triacylglycerol lipase	Performs the first step in TAG lysis		Decreased	leads to steatosis		[58, 59]
*CGI-58*	Gene identification-58	ATGL coactivator			leads to steatosis		[60]
*SREBP-2*	Sterol regulatory element binding protein 1c	Induces cholesterol synthesis		Decreased			[23, 61]
*LXR*	Liver X receptor	Induces cholesterol secretion and FA synthesis, induces CD-36 and Idol	Increased	Increased			[62–64]
*Idol*	Inducible degrader of the LDLR	Idol catalyzes the ubiquitination of LDLR and targets it for degradation	Increased	Increased			[65]
*ACAT2*	Acyl-Coenzyme A: cholesterol acyltransferase	Cholesterol esterification; provides esterified cholesterol for incorporation into VLDL and storage			Protection		[66]
*NPC1L1*	Niemann-Pick C1-like 1	Cholesterol absorption			Protection		[67, 68]

## Abbreviations

ACAT: Acyl-coenzyme A cholesterol acyltransferase
ACC: Acetyl-CoA carboxylase
CETP: Cholesteryl ester transfer protein
CD-36: Cluster differentiation protein-36
ChREBP: Carbohydrate regulatory element binding protein
DGAT2: Diacylglycerol acyltransferase 2
ELOVL6: Family member 6, elongation of long-chain fatty acids
FASN: Fatty acid synthase
HL: Hepatic lipase
HSL: Hormone-sensitive lipase
IDL: Intermediate-density lipoprotein
Idol: Inducible degrader of the LDLR
LPL: Lipoprotein lipase
LDL: Low-density lipoprotein
LDLR: LDL receptor
L-PK: Liver-type pyruvate kinase
LXR: Liver X receptor
MTTP: Microsomal triglyceride transfer protein
NAFLD: Non-alcoholic fatty liver disease
NASH: Non-alcoholic steatohepatitis
PCSK9: Proprotein convertase subtilisin/kexin type 9
SCD1: Stearoyl-CoA desaturase
SREBP: Sterol regulatory element binding protein
TNF-*α*: Tumor necrosis factor *α*
VLDL: Very-low-density lipoprotein.
